# Establishing a baseline of science communication skills in an undergraduate environmental science course

**DOI:** 10.1186/s40594-021-00304-0

**Published:** 2021-07-23

**Authors:** Rashmi Shivni, Christina Cline, Morgan Newport, Shupei Yuan, Heather E. Bergan-Roller

**Affiliations:** 1 Department of Biological Sciences, College of Liberal Arts and Sciences, Northern Illinois University, 447 Montgomery Hall, DeKalb, IL 60115 USA; 2 Weinberg College of Arts and Sciences, Northwestern University, Evanston, IL USA; 3 Department of Communication, Northern Illinois University, DeKalb, IL USA

**Keywords:** Science communication, Environmental science, Undergraduate, Content analysis, Baseline skills

## Abstract

**Background:**

Seminal reports, based on recommendations by educators, scientists, and in collaboration with students, have called for undergraduate curricula to engage students in some of the same practices as scientists—one of which is communicating science with a general, non-scientific audience (SciComm). Unfortunately, very little research has focused on helping students develop these skills. An important early step in creating effective and efficient curricula is understanding what baseline skills students have prior to instruction. Here, we used the Essential Elements for Effective Science Communication (EEES) framework to survey the SciComm skills of students in an environmental science course in which they had little SciComm training.

**Results:**

Our analyses revealed that, despite not being given the framework, students included several of the 13 elements, especially those which were explicitly asked for in the assignment instructions. Students commonly targeted broad audiences composed of interested adults, aimed to increase the knowledge and awareness of their audience, and planned and executed remote projects using print on social media. Additionally, students demonstrated flexibility in their skills by slightly differing their choices depending on the context of the assignment, such as creating more engaging content than they had planned for.

**Conclusions:**

The students exhibited several key baseline skills, even though they had minimal training on the best practices of SciComm; however, more support is required to help students become better communicators, and more work in different contexts may be beneficial to acquire additional perspectives on SciComm skills among a variety of science students. The few elements that were not well highlighted in the students’ projects may not have been as intuitive to novice communicators. Thus, we provide recommendations for how educators can help their undergraduate science students develop valuable, prescribed SciComm skills. Some of these recommendations include helping students determine the right audience for their communication project, providing opportunities for students to try multiple media types, determining the type of language that is appropriate for the audience, and encouraging students to aim for a mix of communication objectives. With this guidance, educators can better prepare their students to become a more open and communicative generation of scientists and citizens.

**Supplementary Information:**

The online version contains supplementary material available at 10.1186/s40594-021-00304-0.

## Introduction

Scientists engage in a number of practices in their pursuit of understanding. Having students participate in these same practices—and as early as possible—is vital in fostering future generations of scientists and developing a scientifically literate society (ACARA, 2012; American Association for the Advancement of Science, 2011; American Chemical Society, 2015; Joint Task Force on Undergraduate Physics Programs, 2016; NGSS Lead States, 2013). One such practice is effective science communication.

Science communication can take many forms and is typically grouped into one of two types depending on the target audience—either a scientific audience or a non-scientific, general audience. While both types of audience-oriented communication are important for scientists and students, the focus of this study is on communicating science with non-experts (abbreviated as SciComm). In the current study, we describe SciComm as the use of appropriate media, messages, or activities to exchange information or viewpoints of science opinion or scientific information with non-experts. Depending on the goal of SciComm, it can be used for “fostering greater understanding of science and scientific methods or gaining greater insight into diverse public views and concerns about the science related to a contentious issue” (National Academies of Sciences, Engineering, 2017a, p. 14).

SciComm is an important scientific practice that benefits both scientists and the public. With effective SciComm, the public learns about foundational and modern scientific understanding that can guide personal and societal decisions. Additionally, the public can appreciate the credibility of scientists and the scientific process to trust scientific consensus even if the scientific content is not easily understood. Communication also allows scientists to recruit more people to engage with science as well as to collaborate and learn about issues in need of more research.

As such, scientists are being encouraged to engage in SciComm by their scientific communities and the public (Cicerone, 2006; Department of Science and Technology, 2014; European Commission, 2002; Jia & Liu, 2014; Leshner, 2007; National Research Council (U.S.). Committee on Risk Perception and Communication, 1989; Royal Society (Great Britain) & Bodmer, 1985), as well as combat the spread of misinformation (Scheufele & Krause, 2019). Additionally, surveyed scientists report viewing themselves as important components in societal decision-making (Besley & Nisbet, 2013) and commonly communicate with the public (Hamlyn et al., 2015; Rainie et al., 2015). Moreover, support and focus for more effective SciComm across STEM fields has grown. For example, researchers have investigated how to communicate engineering issues and technological perspectives of science, such as genetic engineering (Blancke et al., 2017; Kolodinsky, 2018), nanotechnology (Castellini et al., 2007), and artificial intelligence (Nah et al., 2020).

A pertinent example of scientists practicing effective SciComm was seen throughout the severe acute respiratory syndrome coronavirus 2 (SARS-CoV-2) pandemic, where technical experts in virology, epidemiology, data science, etc. took to social media and news media to produce and disseminate evidence-based, accurate health protocols and information about the novel coronavirus (American Society for Biochemistry and Molecular Biology (ASBMB), 2020). During major events, such as the pandemic, scientists are responsible for an important role in communicating emerging science with the public to ease fears, inform decisions, encourage engagement, and give hope to the future.

Because SciComm is an important practice for scientists, it is also essential that undergraduate science students engage with SciComm (Brownell et al., 2013b). All college students are expected to become proficient in interpersonal skills, including communication (National Academies of Sciences, Engineering, 2017b), and this is expressly true for students in STEM fields including biology (American Association for the Advancement of Science, 2011), chemistry (American Chemical Society, 2015), physics (Joint Task Force on Undergraduate Physics Programs, 2016), engineering (Eichhorn et al., 2010; Riemer, 2007), technology (Bielefeldt, 2014), and math (Saxe & Braddy, 2015).

Environmental science is an important context in which to study SciComm skills because it is transdisciplinary—at the intersection of biology, chemistry, physics, and social sciences. Seminal documents in biology (American Association for the Advancement of Science, 2011; Clemmons et al., 2020), chemistry (American Chemical Society, 2015), and physics (Joint Task Force on Undergraduate Physics Programs, 2016) have explicitly stated the need for helping students develop science communication skills. These seminal documents are being used across the sciences to inform curricula and are relevant in guiding curricula and research in environmental science education. Additionally, environmental science encompasses some vital topics relevant to all of society (e.g., climate change) and thus students learning about these important topics should also be learning about how to share that information with the public. Helping a wide range of students develop science communication skills may help students understand scientific concepts, the process of science, and the skills to engage with science after they are out of school regardless of whether they pursue science-related careers. These outcomes are essential in promoting the science literacy of our students and citizens.

### Conceptual framework

**Fig. 1:**
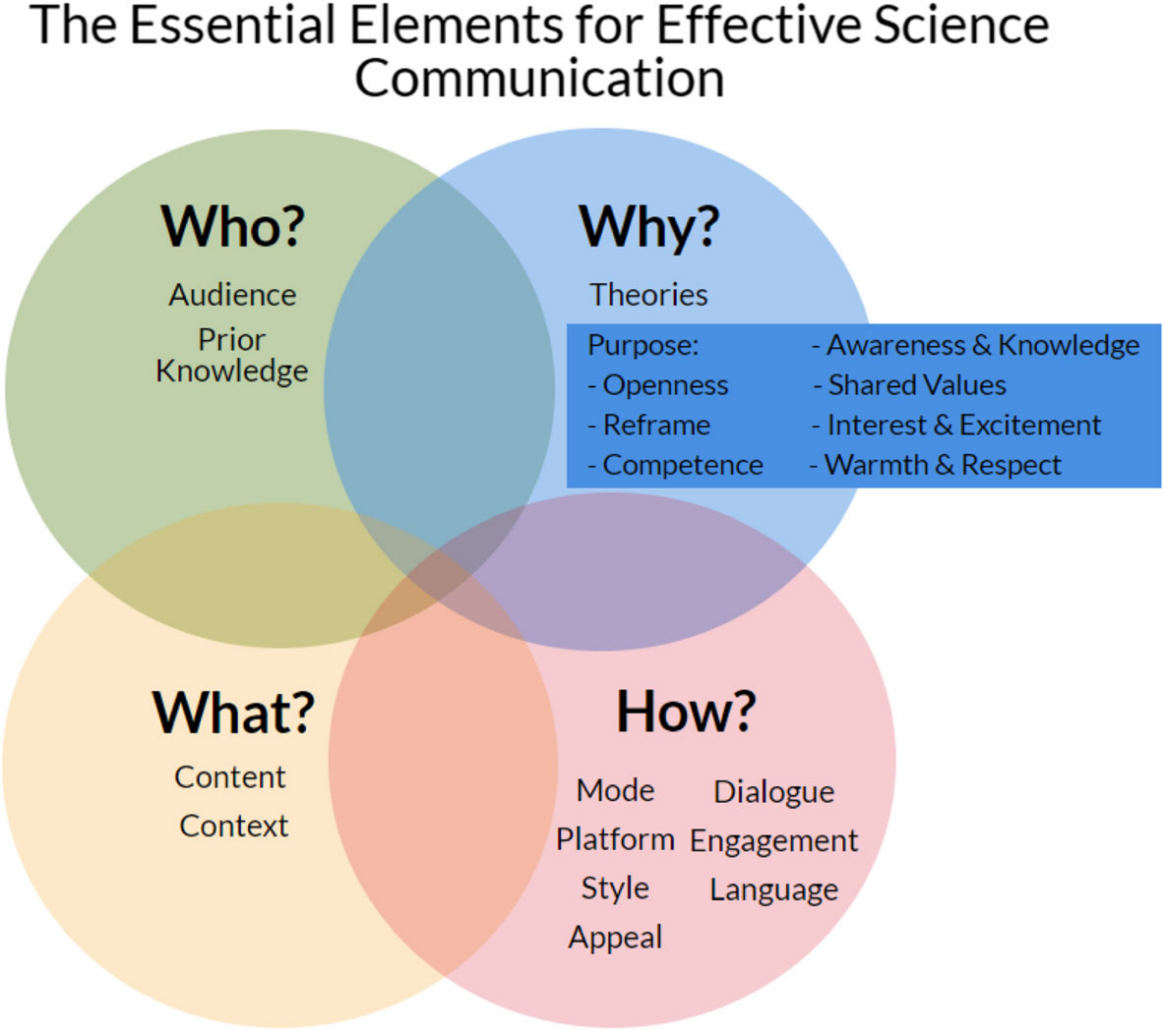
Overview of the Essential Elements for Effective Science Communication (EEES) framework (adapted from Wack et al., 2021). Elements are organized into interrelated strategic categories of who, why, what, and how. The element of purpose is broken down into important SciComm objectives as defined by Besley et al. (2018)

When aiming to help students develop skills, it is an important first step to operationalize those skills. In the context of undergraduate life sciences, the 2011 *Vision and Change* report broadly defined the skills, labeled as core competencies, students should develop in their undergraduate programs (AAAS, 2011). Clemmons et al. (2020) unpacked these core competencies into program- and course-level outcomes. Regarding communication, they define that students should be able to “share ideas, data, and findings with others clearly and accurately”; “Use appropriate language and style to communicate science effectively to targeted audiences (e.g., the general public, biology experts, collaborators in other disciplines)”; and “Use a variety of modes to communicate science (e.g., oral, written, visual).” We expanded those definitions, using evidence-based practices and principles of science communication, to define the key elements of SciComm that are appropriate for undergraduate science students. The resulting Essential Elements for Effective Science Communication (EEES) framework (Wack et al., 2021) adapts skills and concepts from the literature (Besley et al., 2018; Mercer-Mapstone & Kuchel, 2017) and organizes them into four strategic categories of storytelling: “who,” “why,” “what,” and “how” (Fig. 1). The full framework is available in Wack et al. (2021).

The framework is further broken down into 13 elements that are organized under these four categories, which we used to assess the students’ baseline SciComm skills. As shown in Fig. 1, the four categories overlap to represent the interrelated nature of the 13 elements. In order to create effective and cohesive SciComm, each element must be considered in relation to the others. Briefly, we describe the categories and the elements they encompass below.

The elements for *who* science students should communicate science with include identifying and understanding a suitable target audience and considering the levels of prior knowledge in the target audience. The elements for *why* science students should communicate science include identifying the purpose and intended outcome of the communication; this element is expanded upon by the important SciComm objectives defined by Besley et al. (2018)—including to increase knowledge and awareness, boost interest and excitement, listen and demonstrate openness, prove competence, reframe issues, impart shared values, and convey warmth and respect. Further, science students should understand the theories of science communication and why science communication is important. The elements of *what* science students should communicate include focusing on narrow, factual content and situating that content in a relevant context that is sensitive to social, political, and cultural factors. Finally, the elements for *how* science students should communicate science includes encouraging a two-way dialogue with the audience, promoting audience engagement with the science, using appropriate language, choosing a mode and platform to reach the target audience, and adding stylistic elements (e.g., humor, anecdotes, analogies, metaphors, rhetoric, imagery, narratives, and trying to appeal to multiple senses). See Wack et al. (2021) for the full framework.

The EEES framework was originally used to guide the development of a lesson for undergraduate biology students in an introductory lab (Wack et al., 2021). This framework is relevant here because, while biology is only a portion of the course context in this study (i.e., environmental science), this framework was developed to be broadly applicable to any science students in undergraduate programs. Also, the framework describes the best practices for communicating science; through the lens of the backward design process (Wiggins & McTighe, 2005), these best practices can be thought of as learning objectives. Therefore, it is appropriate to then assess student work with the same framework.

### Baseline skills

After operationalizing competencies to provide a clear picture of what instructors should help their students attain, it is also important to understand what baseline skills students have at the start of a lesson; that way, a curriculum can be tailored to skim through honed skills and emphasize weaker skills. Identifying baseline skills, therefore, makes helping students learn these skills as efficiently and effectively as possible (Novak, 2010; Quitadamo & Kurtz, 2007). A similar argument is well-established in the context of helping students achieve conceptual understanding with the literature on *prior knowledge* (e.g., Ausubel, 2012; Bergan-Roller et al., 2018; Binder et al., 2019; Lazarowitz & Lieb, 2006; National Research Council (U.S.) & Committee on Programs for Advanced Study of Mathematics and Science in American High Schools., 2002; Tanner & Allen, 2005; Upadhyay & DeFranco, 2008); however, assessing *skills* before a lesson is less commonly discussed in the literature, which we designate as *baseline skills*.

Assessment is required to identify students’ skills, including their baseline skills. However, to our knowledge, there is very little literature that provides insight into the assessment of undergraduate science students on science communication skills. Kulgemeyer and Schecker (2013) examined how students communicate science in the limited context of older secondary students communicating physics phenomena to younger students. In another study, Kulgemeyer (2018) went further by testing older secondary students on audience-oriented SciComm best practices and found that those with more SciComm experience, or more developed baseline skills, were better at discerning an audience’s needs for particular SciComm content than students who had less experience with SciComm but were quite knowledgeable about the content. Other studies related to students and SciComm have measured application of SciComm knowledge with closed-response quiz questions (Wack et al., 2021), perceptions and confidence in communicating science (Brownell et al., 2013a), the value of SciComm (Edmondston et al., 2010a), and perceptions of SciComm skills (Yeoman et al., 2011); but they have not assessed how students demonstrate SciComm skills. More work needs to be done to assess how students communicate science in a variety of contexts (e.g., disciplines, audiences, level of the student) in order to establish a generalized baseline of skills from which to build an effective curriculum.

In this descriptive study, we surveyed baseline SciComm skills of students in an undergraduate environmental science course in order to inform instructors and curriculum designers on how to help similar science students develop SciComm skills. We took an exploratory, qualitative approach to investigate the following research questions:

- RQ1- How did these students demonstrate their SciComm skills according to the EEES framework?
- RQ2- How did the way these students planned their SciComm compare to how they executed their SciComm projects?
- RQ3- Did instructions influence the SciComm skills that these students demonstrated?

## Methods

We conducted an exploratory case study according to VanWynsberghe and Khan (2007); our unit of analysis was students’ SciComm skills and our case was one undergraduate environmental science course in which the students demonstrated their baseline skills with a project that included planning and executing a SciComm product.

### Study context

The study was conducted at a large 4-year, doctoral-granting, regional comprehensive university in the Midwestern United States with students enrolled in an environmental science course. This course focused on the functioning of ecosystems, the patterns of biological diversity, the processes that influence those patterns over space and time, and how human activities can disrupt those processes. The course included a SciComm project, which we used for this research; however, SciComm was not a focus of the course. Students did not receive formal training on the underlying theories or practices of SciComm relevant to the EEES framework or otherwise; and we did not gather background information on whether students had knowledge from elsewhere to apply to their SciComm projects. We saw this as a unique opportunity to obtain a baseline of SciComm skills.

Study participants were recruited by one author attending a class period early in the semester, describing the study, and asking for their explicit consent. The entire class was given the opportunity to participate in the study, of which 32 (65%) consented. Students were assigned to plan and execute SciComm products, which we analyzed for this research. From the consenting students, 27 plans and 21 products were available for this research. All names listed herein are pseudonyms. Demographics for each of these populations are shown in Table 1 and the result show that they are equivalent. Generally, the samples consisted of more females than males. Most of the students were White/non-Hispanic, juniors, and 18–25 years old. About one-third of the students were first-generation college students and two-thirds were transfer students. Cumulative GPAs averaged 3.1 to 3.3 (with standard deviations of 0.9). The demographics of these students are typical for the university and major, as well as for undergraduate biology students throughout the USA—as compared to data from the U.S. Department of Education’s National Center for Education Statistics (Data USA, 2018).

**Table 1:** Demographic information from the consenting students and their coursework (plans and products) included in this research

	Consenting	Plans	Products
*n*	32	27	21
Females	19	18	14
Males	13	9	7
18–21 years	10	10	7
22–25 years	14	12	9
26–30 years	6	4	4
31–40 years	2	1	1
White/Non-Hispanic	28	24	18
Other race/ethnicity	4	3	3
Freshman	1	1	1
Sophomores	6	5	3
Juniors	17	14	12
Seniors	6	5	4
Post-bachelors	2	2	1
First generation	11	9	7
Transfers	21	18	14
Cum. GPA (SD)	3.1 (0.9)	3.2 (1.0)	3.3 (0.9)

Numbers represent students in each category of consenting students and the student plans and products that were available for this research

### Assignment

As a regular part of the course, students were assigned a project to communicate science with a general, non-scientific audience. Their projects included having students submit a plan to the instructor, who gave individual feedback, and then execute their plan in what we call their product. Assignment instructions and rubric, which were provided to the students when the project was assigned, are available in supplemental materials S1 and S2, respectively. Students were given creative freedom to communicate scientific content—using any means such as presentations, social media, and blogging—to a specific audience of their choosing. The instructions required the students to interact with an audience from the public. Though the assignment was developed solely by the instructor (the researchers and the framework were not a part of the assignment design), there was some overlap with the EEES framework that was explicitly mentioned in the assignment.

### Data sources

Several course artifacts and student demographics were collected for this research (Table 1). Students’ plans and products were collected to identify which elements of the framework they included as evidence of their baseline skills. The students’ final products are available through the figshare data repository (Bergan-Roller & Yuan, 2021). Additionally, we collected the assignment instructions and rubric (supplemental materials S1 and S2) to identify which elements of the framework were included in order to provide insight into the possible influence that instruction can have on the students’ demonstration of skills. However, we did not analyze the individualized feedback given by the instructor after students submitted their plans as we focused on students’ skills in aggregate.

### Analysis

**Fig. 2:**
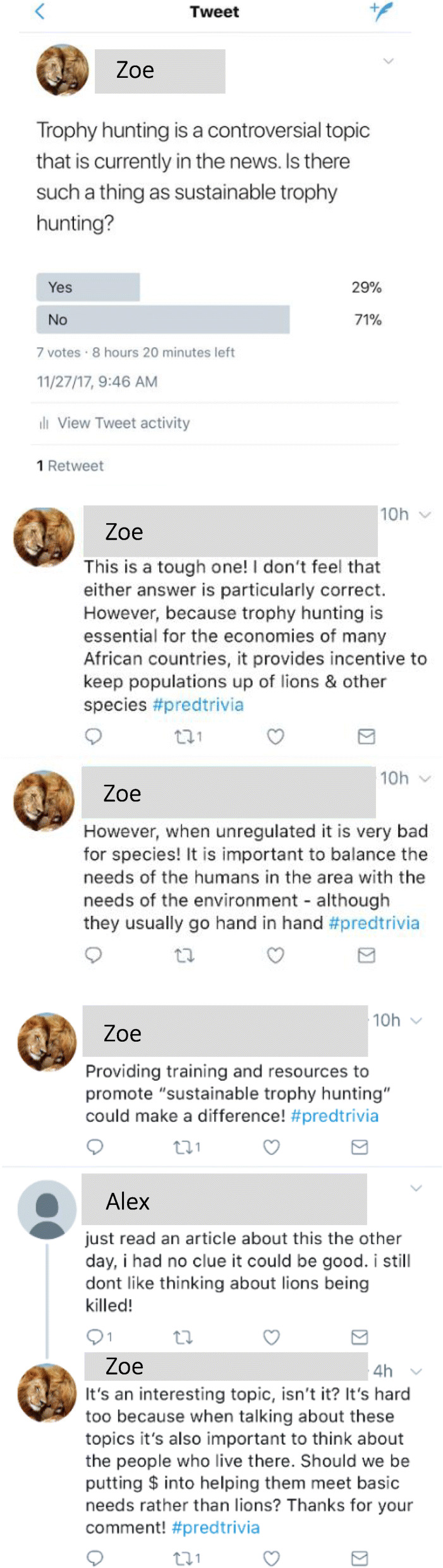
Example product from student Zoe. This product was coded to include the following elements with the types and levels indicated in parentheses: audience (general, primarily young adult to adult), content (apex predators and ecological topic; human and biological components), dialogue (social media Q&A and conversations with audience members; high), language (no jargon, mixed formality), mode (remote location; print media), platform (social media, specifically Twitter), and engagement (asks specific questions; low). The product was absent of style, appeal, and context. The elements of prior knowledge, purpose, and theory were not observable for any products

The plans, products, assignment instructions, and rubric were imported into qualitative software (NVIVO) and analyzed using content analysis which describes the themes in artifacts such as coursework (Neuendorf, 2017). First, we conducted a priori thematic analysis by coding for the presence or absence of each of the elements of the EEES framework (codebook provided in Supplemental Materials S3). Three elements were not observable in the products (purpose, prior knowledge, and theory). After the presence of elements was identified, student plans and products underwent further thematic analysis to identify themes in how students addressed the elements of the framework (Braun & Clarke, 2006). An excerpt of an example product is presented in Fig. 2 with a description of how it was coded in the figure caption. To ensure the reliability of the codes, two of the authors co-coded all the data. The initial agreement was 83%. All dissimilar codes were discussed to a consensus, and the codebook was revised to clarify the codes. The final codebook is available in supplemental materials S3.

Most students completed the assignment individually; however, when a pair worked together on the assignment, the project artifacts (plans and products) were treated as single artifacts. This work was conducted with prior approval from the institutional review board (#HS17-0259).

## Results

Below we describe if and how the elements of the EEES framework appeared in students’ projects (i.e., plans and products). Later, in the discussion, we interpret these descriptions to characterize these students’ baseline SciComm skills. Additionally, we examined the project instructions for alignment with the EEES framework as an indication of how instruction may be able to influence the development of SciComm skills in undergraduate science students.

### Presence of SciComm elements

The elements of SciComm that students described in their plans were similar to those demonstrated in their products, but there were a few key differences (Table 2). Students described a similar number of elements in their plans (8.0 ± 1.0) as they demonstrated in their products (8.1 ± 0.9), despite all 13 elements being observable in plans but only 10 being observable in products. Most to all the students described the elements of content, platform, mode, audience, dialogue, and engagement in their plans and demonstrated these elements in their products. Additionally, plans and products were similar in how few students included the elements of context and style. Dissimilarities existed in the number of students who described intending to use language in the plans and who demonstrated language in the products. Appeal was also present in more products than plans. Most students described a purpose in their plans while less than a third described considering the prior knowledge of their audience or the theoretical rationale for their decisions.

**Table 2:**
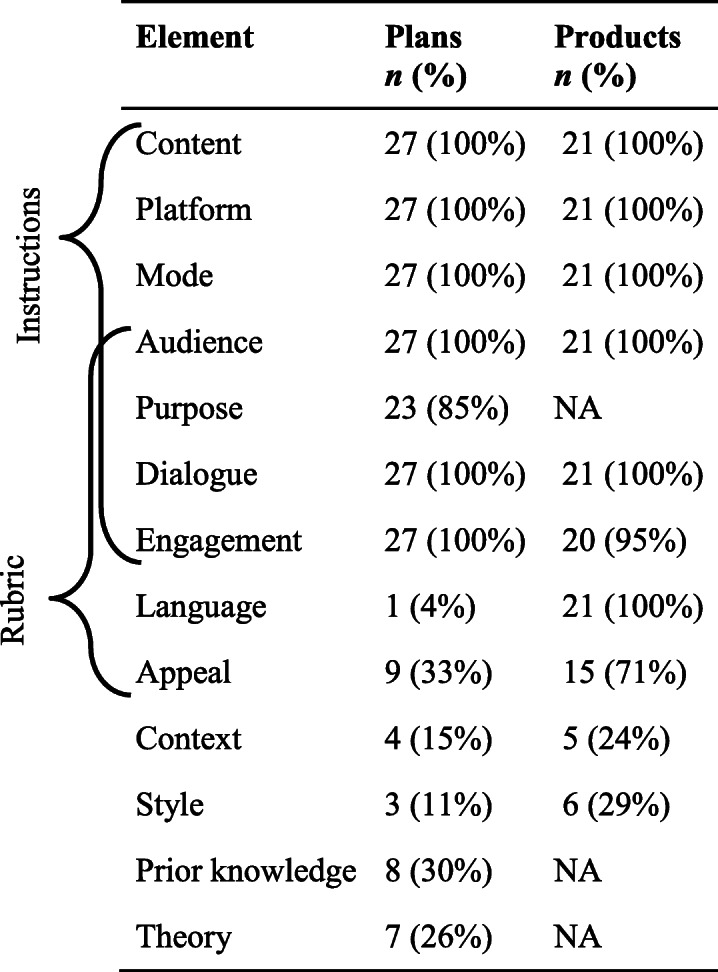
Presence of essential elements for effective SciComm in student projects out of 27 plans and 21 products

Elements that were not observable are denoted with NA. Brackets in the left margin indicate which elements were explicitly addressed in the assignment instructions and rubric

The instructor’s assignment instructions and rubric included some of the EEES framework elements even though the instructor did not have the framework and the researchers did not direct the instructor on assignment design prior to the semester. Nevertheless, we compared what elements appeared in the assignment instructions and rubric with the elements students demonstrated in their projects to provide insight into the effect that instruction can have on the students’ demonstration of skills (as further explained in the discussion). Elements that were explicitly mentioned in the assignment instructions were described in plans and demonstrated in products by most students (Table 2); fewer students described elements in their plans that were only present in the rubric, while many more students demonstrated these rubric-only elements in their products. Elements that were not explicitly asked for in either the instructions or rubric were present in the fewest student plans and products.

### Themes for how students presented SciComm elements

Beyond if the elements were present in the students’ projects, we analyzed *how* the students presented these elements. We organized the results below into the four strategic categories to which the elements belong in the framework.

#### Who did students communicate with?

##### Audience

The students defined their audiences through categories of specificity, age, and interest (Table 3). More than half the students targeted both a specific audience in conjunction with a general audience in their plans and products. For example, Wells wrote,

**Table 3:** Thematic categories and subcategories of students’ target audiences out of 27 plans and 21 products

Audience	Plans *n* (%)	Products *n* (%)
Specificity	27 (100%)	21 (100%)
Specific	25 (93%)	19 (90%)
General	18 (67%)	15 (71%)
Age	19 (70%)	9 (43%)
Adult	11 (58%)	5 (56%)
Young Adult	8 (42%)	2 (22%)
Child	5 (26%)	3 (33%)
Interest	15 (56%)	NA
Interested	13 (87%)	NA
Uninterested	4 (27%)	NA

Numbers represent the number of students that defined their audience with each category (i.e., specificity, age, or interest) and subcategory. Percentages represent the percent of students that described their audience with the subcategory (e.g., adult) out of the number of students that defined their audience within the broader category (e.g., age)

My target audience would be people that work outdoors first and foremost, as this issue would affect them the most from a health perspective. Otherwise, I think the environmental aspect of the issue affects everyone and anyone, so I would want to spread that information to as many people as possible.

When specifying their audience, the students described age and interest. More students targeted adults over young adults or children. In the plans, about half of the students aimed for an audience with identified interest or non-interest in the scientific content that they intended to communicate. Of the 15 plans that addressed the interest of the audience, most targeted an audience with an interest in the subject. A few of the students explicitly sought out an audience who were not already interested in the scientific content (Table 3). For example, Bellamy wrote,

I hope to reach people that are not extremely in tune with the environment.

Two out of the 27 plans (Bellamy and Echo) described wanting to address an audience that included both interested and uninterested members. The interest of the audience was not observable in the final products as this work focused on the students and their work, not the students’ audiences.

##### Prior knowledge

The students approached the element of prior knowledge by collecting and sometimes using information about their audiences’ understanding to influence their projects. Eight students (30%) planned to collect information on the prior knowledge of their audience. For example, Raven wrote,

I plan to ask the children about their own thoughts on the subject, of what they already know about sharks and how they perceive them, why they think sharks are important and helpful to the ecosystem, and what they can do to help preserve the shark's habitat.

Raven planned to move forward with her presentation irrespective of the children’s input. Four students (15%) described planning to use the prior knowledge information they gathered by adapting their products accordingly. For example, Niylah wrote that she would (emphasis is ours):

create a survey with a mixture of multiple-choice and open-ended/extended-response questions to gauge the public’s knowledge on recycling (what is recyclable, where do these materials go after they are recycled, etc.) and what questions they have about recycling…Create easy-to-understand and visually appealing infographics on recycling **based on survey results**…in an attempt to address and clarify common misconceptions.

#### Why did the students communicate this science?

##### Purpose: communication objectives

We examined how students described the purpose of their projects in their plans through the lens of Besley’s work that defines important science communication objectives (Besley et al., 2018) (Table 4). Several students intuitively developed their project’s purpose and described between zero and four objectives with two objectives being the most common (9 students, 33%). The objective to increase knowledge or awareness was the most common followed by the explicit goal to cause their audience to act, which is not a part of the Besley framework of objectives. For instance, Wells planned to create a public service announcement to show the effects of climate change on human health. His call to action was to help people slow the buildup of greenhouse gases from everyday changes, such as providing examples of cleaner forms of transportation and energy use.

**Table 4:** Science communication objectives students reported as the purpose of their projects out of 27 plans analyzed through the work by Besley et al. (2018)

Purpose objective	Plans *n* (%)
Increase knowledge and awareness	21 (78%)
Other: take action^a^	12 (44%)
Boost interest and excitement	10 (37%)
Listen and demonstrate openness	7 (26%)
Reframe issue	4 (15%)
Convey competence	2 (7%)
Convey warmth and respect	2 (7%)
Convey shared values	2 (7%)

^a^Not present in the Besley framework but emerged from our data. Objectives were not observable in products

The next most common objectives were to boost interest and excitement, as well as listen and demonstrate openness. For example, Echo demonstrated openness by starting a discussion on Facebook—within her circle of family and friends—to understand different points of view on climate change. She stated that she would “respond politely with facts, but in a way where [my peers] don’t feel attacked.” Few students included any one of the other four objectives.

##### Theory

For the students that included some element of theory (7 plans, 26%), their rationalization for why they made certain decisions did not align with science communication theory or evidence-based practices. For example, Clarke said she wanted to make the project entertaining so that the audience would be more likely to remember the information, and Anya chose college students as a target audience because she believed that people who go to college are more passionate and generally interested in changing the world. These explanations seemed to be based on their interpretations of how learning works and how education increases interest, respectively, but not necessarily based on the literature.

Another student, Madi, chose a target audience of high school students because “They are mature enough to instill the information being taught, but just as immature enough to refuse to accept it.” Her rationale stems from, as she explained, her upbringing in a household with parents who were teachers. Though not established in the literature on teaching nor SciComm, this student made a decision about her audience based on descriptions from her parents—her authority figures.

#### What did the students communicate?

##### Content

We analyzed the scientific content of the students’ projects regarding what components they included and what topics they focused on (Table 5). Most to all students incorporated a human component to their projects and several included a biological (non-human) component. The human component was labeled if the plans and products presented anything related to human involvement. For instance, climate change would fall into this category only if a student explicitly talked about human roles in either causing climate change or how their actions could mitigate the effects of climate change. There had to be some language explicitly relating to people and not just assumed human involvement. For the biological component, the projects had to explicitly reference non-human biological species. For example, a student working on a climate change SciComm project would need to mention the effects on other species than humans. Components relating to earth sciences (e.g., weather and oil spills) were present but infrequent (four or fewer students). The students focused on topics that were covered at other times during the course at relatively equal proportions with an ecological topic being slightly more popular than sustainability or climate change.

**Table 5:** Thematic categories and subcategories of content out of 27 plans and 21 products

Content	Plans *n* (%)	Products *n* (%)
Components		
Human	27 (100%)	20 (95%)
Biological	13 (48%)	15 (71%)
Topics		
Ecology	10 (37%)	10 (48%)
Sustainability	9 (33%)	6 (29%)
Climate change	6 (22%)	6 (29%)
Other	2 (7%)	NA

Numbers represent the number of students that included a biological or human component or focused on the listed topics

##### Context

Some of the students considered the social, political, and/or cultural context of the scientific information (4 out of 27 plans, 5 out of 21 products). Although there were too few of these students to decipher themes within context, examples included describing the culture of coastal fishermen in relation to overfishing issues (Harper), and that the ability to choose foods from sustainable farming practices may be impacted by socioeconomic status (Lincoln).

#### How did the students communicate science?

##### Dialogue

Dialogue pertains to any conversation between the student presenter and the audience. Conversation could be on any subject including on scientific content being communicated or other topics. Student plans and products were analyzed for the element of dialogue in two ways: the direction and level of dialogue. For the direction of dialogue, all students talked to their audience and most students also received input from their audience (Table 6).

**Table 6:** Thematic categories of how students communicated, including dialogue, engagement, language, mode, and platform out of 27 plans and 21 products

Element	Theme	Plans ***n*** (%)	Products ***n*** (%)
**Dialogue**	Direction		
	Student to audience only	7 (30%)	2 (10%)
	Audience to student only	0	0
	Both	20 (74%)	19 (90%)
	Level		
	Low	7 (26%)	2 (10%)
	Medium	16 (59%)	7 (33%)
	High	4 (15%)	12 (57%)
**Engagement**	Type		
	Passive	23 (85%)	11 (52%)
	Questioning		
	From student	9 (33%)	14 (67%)
	From audience	14 (52%)	18 (86%)
	Active	1 (4%)	1 (5%)
	Ambiguous	3 (11%)	NA
	Level		
	Low	8 (30%)	4 (19%)
	Medium	12 (44%)	9 (43%)
	High	7 (26%)	8 (38%)
**Language**	Jargon		
	Use	0	8 (38%)
	Not use	1 (4%)	13 (62%)
	Formality		
	Only formal	0	4 (19%)
	Only informal	0	8 (38%)
	Mixed	0	9 (43%)
**Mode and platform**	Location		
	Remote	19 (70%)	14 (67%)
	In person	9 (33%)	8 (38%)
	Media type		
	Print	7 (26%)	13 (62%)
	Audio	13 (48%)	2 (10%)
	Video	10 (37%)	6 (29%)
	Social media		
	Use	19 (70%)	14 (67%)
	Not use	8 (30%)	6 (33%)

Numbers represent the number of students under each subcategory

The level of dialogue indicated how much dialogue was planned or occurred. Low dialogue was when only one direction of communication was planned or occurred (e.g., student communicating to the audience only). Fewer students executed low dialogue than described low dialogue in their plans (Table 6). Medium dialogue was when both directions of dialogue were planned or occurred, but one direction was much more prevalent than the other (e.g., a presentation with a brief question-and-answer (Q&A) session). Over half of the students described medium dialogue in their plans while only about a third executed dialogue at this level (Table 6). High dialogue was when both directions of dialogue were planned or occurred frequently and throughout the communication. The fewest number of students planned high dialogue, although the largest number of students executed high dialogue (Table 6).

##### Engagement

Engagement pertains to how the audience engages with the science. Student plans and products were analyzed for the element of engagement in two ways: the type and level of engagement. Most of the students passively engaged their audience by having the audience listen and/or observe the presentation (Table 6). Engagement commonly took the form of asking the audience specific questions about the science or allowing for questions or comments from the audience. Only 1 out of 27 students planned to actively engage their audience with the science by having them play a board game on migration and go bird watching (Indra). Only 1 out of 21 students executed active engagement by having students identify rocks with a game (Lexa). A few of the students mentioned engaging their audience with the science but did not further describe how they planned to do so (coded as ambiguous) (Table 6).

The level of engagement indicated how much the student planned or facilitated the audience to engage with the science. Low engagement was when the student presented to the audience who only viewed or listened nearly the entire time. A third of students planned to engage their audience at a low level but a slightly lower percentage executed low-level engagement (Table 6). Medium engagement was when the student presented and the audience viewed and/or listened most of the time but there were some other types of engagement, commonly as questions between the audience and student. Most students planned and executed medium-level engagement (Table 6). High engagement was when the student facilitated active and/or frequent engagement between the audience and the science, such as the audience answering frequent specific questions and modeling or observing a scientific phenomenon (e.g., bird watching or the rock game). The fewest students planned high-level engagement; however, more of the students executed high engagement (Table 6).

##### Language

We coded language for whether students used jargon and the formality of their language (Table 6). Only 1 out of the 27 students (Abby) described in her plans what language she would use by “avoiding jargon.” More students omitted jargon from their products than included jargon. More students used informal language when communicating science than formal language, or they used a mix of formal and informal rhetoric.

##### Mode and platform

The students approached the elements of mode and platform in terms of location, use of media types, and use of social media (Table 6). More of the students had projects that were remote from their audience than in-person. A few of the students planned projects that involved both remote and in-person portions. In-person projects were commonly set in a classroom. As for media types, most students used print media (e.g., the Twitter Q&A and conversations in Fig. 2) in their final products and several students used multiple types of media (Table 6). While many of the 27 students planned to do audio-based projects such as podcasts, only 2 out of 21 executed that plan. Regarding where to put their SciComm, most students included social media, which included sites like Facebook, Twitter, and YouTube (Table 6).

##### Appeal and style

The students appealed to their audiences’ senses primarily with visuals including PowerPoint slides, photos, artwork, and charts. Some of the students used stylistic elements to present scientific information. For example, Bellamy included humor and satire by dressing up in a penguin suit and advertising to “kill the penguins.” Gaia employed narration and described her adventures at the local farmer’s market.

## Discussion

To tailor a curriculum to be meaningful and authentic, educators and education researchers need to first define learning outcomes that align with professional, scientific practice, and then use those definitions to assess students’ baseline skills, including for SciComm. Then, the curriculum can be built upon this solid foundation. Here, we provided a rich description of the baseline SciComm skills of students in an undergraduate environmental science course. Overall, our results showed that these undergraduate students are on their way to being effective science communicators and have room to develop these skills further with proper curricular support. We next interpret that description to guide instructors on how to help students develop important SciComm skills.

Students demonstrated their skills consistently, between their plans and products, in many ways including identifying their audience and focusing on factual content. However, there were a few notable exceptions. Students planned primarily one-way dialogue (e.g., talking at a class) but executed frequent two-way dialogue (e.g., played a game with the audience) throughout their SciComm; this switch to more interaction from planning to execution was similar to how students engaged their audiences with the science. But not all skills listed in the framework were observed in the students’ work, which provides instructors the room to give students a wide variety of opportunities and circumstances to demonstrate, practice, and develop their SciComm skills.

Furthermore, the results showed that it is important to recognize the value of the instruction given by the instructor, which affected the types of skills students demonstrated. The students demonstrated most of the elements in their plans and products that aligned with what was asked of them in the instructions. This suggests that students would benefit from explicit SciComm instruction and training on effective SciComm to develop their SciComm skills in the context of their science coursework.

### Pedagogical and curricular recommendations for integrating SciComm into science courses

Below, we take a fine-grain view of the SciComm skills these students demonstrated and make recommendations on how instructors and curriculum can build off this baseline to effectively help science students develop their SciComm skills.

#### With whom to communicate science

Help students identify a narrow audience. Our findings showed that the students commonly described a specific population but then also described trying to reach a broader audience. Students may need help recognizing that fostering quality communication with a small and specific audience is more effective than just exposing their SciComm to large quantities of people (Mercer-Mapstone & Kuchel, 2017).

Help students understand their audience. Here, about a third of the students considered the prior knowledge of their audience and fewer used it to influence their products. Similarly, about half of the students did not describe whether they thought their audience was explicitly interested or not interested in the subject. A presenter must acknowledge and understand what their audience already knows (i.e., prior knowledge) and what the audience is interested in to increase knowledge (Ausubel, 2012; Novak, 2010; Vosniadou, 2013), which was the most commonly stated purpose objective. This is true whether the setting is a classroom between an instructor and students or on a public stage such as with these environmental science students and their target audiences.

#### Why communicate science

Introduce students to the theories that make for effective SciComm. Despite not being asked to, some of the students described their rationale behind why their project would effectively communicate science with the public (theory element). However, these explanations seemed to be based on intuition, and were lacking operational description, which are often ineffective and can be harmful to the public’s perceptions of science (Scheufele, 2013). Therefore, instructors may consider introducing SciComm via its theoretical underpinnings to help students better understand the need for developing such skills.

Encourage students to aim for diverse communication objectives. Here, many students intuitively aimed to increase knowledge and awareness. Similarly, scientists focus more on this traditional knowledge-based objective than other equally important objectives (Besley et al., 2018). Nevertheless, scientists, and thus science students, need to aim beyond just increasing knowledge and awareness as many other objectives are key to effective SciComm (Besley et al., 2018). Specifically, appropriate for science students are the objectives of boosting interest and excitement, conveying warmth and respect, conveying shared values, and listening and demonstrating openness (Fig. 1). Further, having an audience take action is an assumed, ultimate goal of communication (Besley et al., 2018); here, about half of the students’ plans made this goal explicit. More work is needed to know if students are thinking about an ultimate goal for their SciComm. Together, our work suggests that the curriculum should provide support to help students identify their broader goals and specific objectives for SciComm.

#### How to communicate science

Give students practice with multiple media types. Here, many students planned to use audio and video, but then executed their SciComm with print media. A recent report concluded that Gen Z (people born between the mid-1990s and the mid-2000s) prefer video over print for learning, whereas Millennials (people born in the early 1980s to mid-1990s) prefer print (Pearson Education Inc., 2018). The students studied here were composed of approximately 75% Gen Z and 20% Millennials. One explanation for our results could be that the students had ambitions to increase the knowledge and awareness of their audience using a medium which they themselves prefer and commonly consume (video) but potentially experienced logistical constraints that directed them to a simpler media (print) that could still reach a large audience (e.g., Lincoln’s switch from podcast to print). Scientists have increasingly connected with the public, using print, audio, and video remotely due to the SARS-CoV-2 pandemic (ASBMB, 2020). Therefore, students need practice with a variety of media types, especially on a variety of platforms as communication with the public evolves.

#### Example curricula

There are a few published examples of integrated SciComm and science curriculum that may help science students develop their SciComm skills. These are organized either as whole courses or modules within science courses. Examples of whole courses include an undergraduate neuroimmunology and writing course (Brownell et al., 2013a) and a biotech and SciComm course (Edmondston et al., 2010a, 2010b). Examples of the modular approach have been documented in the contexts of junior high school (Spektor-Levy et al., 2008, 2009), undergraduate physics (Arion, 2016; Arion et al., 2018), mid-level undergraduate biology, physics, and chemistry (Mercer-Mapstone & Kuchel, 2016), and upper-level undergraduate biology (Yeoman et al., 2011). Additionally, we applied the EEES framework to develop and assess a module for introductory undergraduate biology students (Wack et al., 2021). These curricula may be excellent sources for instructors looking for guidance on how to help their students develop SciComm skills.

#### Assessment and feedback

Vital components of learning are assessment and feedback. Assessment of students should be based on the learning goals and objectives that instructors make explicit at the beginning of any lesson (Wiggins & McTighe, 2005) and thus can vary considerably. The options to assess SciComm lessons include what others in the literature have done, including using a closed-response quiz where students apply their knowledge of SciComm (Wack et al., 2021); asking for students to report on their gained skills (Yeoman et al., 2011); measuring perceptions, value, and confidence in communicating science (Brownell et al., 2013a; Edmondston et al., 2010a); and characterizing the skills students demonstrate as we have done here. Additional assessment could include input from the audience to gauge the effectiveness of the communication. These assessment options can be used to provide feedback to students so that they may reflect on their performance and how they may perform better in the future—an important step in developing lasting skills.

### Limitations and future directions

We recognize the limitations of this research and suggest how future studies could augment this work. For instance, we intentionally omitted giving the students the framework in the instructions and rubric so that we could observe a baseline of SciComm skills. Future work should investigate how providing different scaffolds, or support such as the framework, affects students’ SciComm skills.

By using content analysis of student work, we were able to provide rich descriptions of students’ SciComm skills. Future work should use student interviews and reflective journaling to triangulate evidence on SciComm skills. When only a few students described a certain element, it reduced our ability to establish themes for how students commonly address an element and limits the generalizability of the results. Nevertheless, our findings on these elements provide some anecdotal examples of what one might expect from their students or study population.

Many of the elements of SciComm are intertwined, as are best practices for SciComm. For example, the audience one targets (e.g., young children) will impact the platform they choose (e.g., a classroom, not Twitter). These interconnections led to occasional overlap in our coding (e.g., engagement/dialogue, types/levels) and results could be influencing other results. Nonetheless, descriptions of each element provided a comprehensive survey of the students’ baseline skills and thus were important to characterize individually.

We recognize that this is just one class in one context; much more work needs to be done in a variety of contexts, and separate results based on student demographics, to gain additional perspectives on undergraduate life science students’ baseline SciComm skills. For example, repeating this study with larger groups of students in more disciplines would improve statistical strength; additionally, larger samples would allow for testing the effects of age or experience on outcomes so that these results may be extrapolated to other institutions and other disciplinary contexts across STEM fields.

## Conclusions

SciComm is an important scientific practice for which undergraduate science students should develop skills. To effectively help students develop these skills, it is important to understand what baseline skills students have. Here, we used the EEES framework to explore the SciComm skills students in an environmental science course demonstrated with little training. Despite not being given the framework, students included several of the 13 elements, especially those which were explicitly asked for in the assignment instructions. Students exhibited SciComm skills similar to scientists who are novice in SciComm but showed promising development by following many of the instructions and refining their work from planning to execution. Together with the recommendations we make for how instructors can use these findings, a curriculum that is grounded in effective science communication can help undergraduate science students develop meaningful SciComm skills.

## Supplementary Information

**Additional file S1:** Assignment instructions

**Additional file S2:** Assignment Rubric

**Additional file S3:** Codebook

## Data Availability

The datasets used and/or analyzed during the current study are available from the corresponding author on reasonable request. Student products, specifically, are available in the figshare repository, 10.6084/m9.figshare.14544072 (Bergan-Roller & Yuan, 2021).

## Abbreviations

EEES: Elements for Effective Science Communication framework
Plans: Written documents students submitted to plan their SciComm
Products: Evidence students submitted of their executed SciComm
Projects: The combination of students’ plans and products
Q&A: Question and answer
SARS-CoV 2: Severe acute respiratory syndrome coronavirus 2
SciComm: Communicating science with non-experts
