# Supplementary material for: Establishing a baseline of science communication skills in an undergraduate environmental science course
Source: Int J STEM Educ. 2021 Jul 23;8(1):47. doi: 10.1186/s40594-021-00304-0 (PMC8299166; doi:10.1186/s40594-021-00304-0)
Supplement: Supplementary file 1 — Additional file S1: Assignment instructions [file 40594_2021_304_MOESM1_ESM.docx]

**Public Outreach Instrument**

The public outreach instrument (POI) is your time to get creative and to learn to communicate to a non-scientific audience. The POI can be any type of media you think will help get the public engaged in the science of your topic. And you can choose any of the three team-based projects* to communicate to the public about.

Here are potential options for your POI, but feel free to come up with your own outreach as well:

- Video or recording of yourself doing a Public Service Announcement. Make sure you plan to ensure you will get public interaction.
- Pre-plan a day on your favorite social media platform each week to engage with the public through posting and question/answer sessions. Curate it with #ENVS302.
- Do a Facebook Live event and invite your friends and family to chat with you about your group’s topic.
- Do a podcast and interview someone who works on this topic and post it on social media. Make sure you plan to ensure you will get public interaction.
- Do something like Story Corps where you record yourself and someone who may have a differing viewpoint on the topic talking to each other about it and post it on social media.
- Record yourself talking to K-12 students about your topic, and/or guiding them through hands-on activities to help explain it and post it on YouTube.
- Create a piece of art that you post on social media or somewhere on campus with which the public can interact about the topic.

**Note:** If you choose Twitter or Instagram as your POI medium, please use the hashtag #ENVS302. On Twitter, I will retweet your POI and encourage engagement from the public. If you use the hashtag, I will assume I have your permission to amplify your content.

The main goal of the POI is for you to start exploring ways of connecting with the non-scientist community. Because this is 12% of your grade – nearly as much as both tests in the class – I expect a significant amount of effort to be put into this project. Therefore, I will have to approve of your idea for your POI early on so that I know you’ve thought about what you’d like to do and you have a plan to achieve it. I will post your POIs, with your permission, to a common hashtag on Twitter – #ENVS302 – so that my followers can see what you’ve come up with. If you’d like me to tag you, make sure you include your handle somewhere in your submission.

I understand some of you may wish to avoid having your name or work on social media. If that is the case, you may choose to do a Podcast or audio recording which does not identify you or you could do a video of your interaction with the public that is not posted to social media. Please talk to me if you have concerns.

***Two*** ***products are required for the POI:***

1. **Due September 21st on Blackboard before the start of class** – POI Plan. This plan should be no more than 1-2 pages double-spaced and include the following:
2. A short introduction of what you plan to do for your POI.
3. Identify the specific medium you will use for your POI.
4. Identify a specific target audience for your POI. Who are you hoping to reach?
5. A list of goals for your POI. What do you hope to accomplish?
6. A plan for how you will engage and interact with the public. Part of your grade is how much engagement from the public you get and how well you answer inquiries, so you need to make sure you can disseminate your POI in a way that invites dialogue with members of the public.

2. **Due November 30th on Blackboard before the class starts** – POI. Depending on your medium, your POI may be a link to the social media that you posted your POI on, which shows your interactions with the public, or it may be video, audio, or art. If your file is too big to upload to Blackboard, consider posting it to YouTube and submitting the link or using OneDrive to upload and share your file with your TA and me. **Please plan ahead if you have a large file to upload to** **ensure you can share your file via OneDrive without incident.** Because part of your grade is dependent upon interaction with the public, you will need to either record such interactions if they are in person or provide a link to online interactions.

**Specifics of the POI:**

Length will vary significantly depending on the media format chosen. A public service announcement would need to be short (<2 minutes), a video of K-12 outreach would likely be ~30 minutes, a podcast would be ~30 minutes and an interview might be 10-20 minutes.

***Comment on content**:

The instructor indicated the following about the scientific content: "I eventually told them to pick anything they were excited about, so you will be seeing more [content] than just these [three team-based project topics]:

1. Campus sustainability or sustainability more generally

2. Top predators, their impacts on ecosystems, consequences of their decline, and ways to conserve them

3. Human health issues associated with climate change, or climate change more generally."
