# Supplementary material for: Establishing a baseline of science communication skills in an undergraduate environmental science course
Source: Int J STEM Educ. 2021 Jul 23;8(1):47. doi: 10.1186/s40594-021-00304-0 (PMC8299166; doi:10.1186/s40594-021-00304-0)
Supplement: Supplementary file 2 — Additional file S2: Assignment Rubric [file 40594_2021_304_MOESM2_ESM.docx]

| **CATEGORY** | |  | **Excellent- 19.8-22** | | **Good-17.6-19.7** | **Satisfactory-15.4-1** | |  | **Needs Improvement** | |  |
| --- | --- | --- | --- | --- | --- | --- | --- | --- | --- | --- | --- |
|  |  |  |  |  |  | **7.5** | |  | **0-15.4** |  |  |
|  |  |  |  |  |  |  |  |  |  |  |  |
| **Total possible points possible: 110** | | | | |  |  |  |  |  |  |  |
|  |  | | | |  |  |  |  |  | |  |
| **Ideas** | Highly creative. Shows | | | | Creative. Showed |  | Somewhat creative. |  | Not creative. Depth of | |  |
|  | excellent depth of | | | | good depth of thought |  | Showed average depth |  | thought and | |  |
|  | thought and originality. | | | | and originality. Most |  | of thought and |  | originality lacking. | |  |
|  | Any feedback from | | | | feedback from initial |  | originality. Some |  | Very little feedback | |  |
|  | initial idea was | | | | idea was incorporated. |  | feedback from initial |  | from initial idea was | |  |
|  | incorporated. | | |  |  |  | idea was incorporated. |  | incorporated. | |  |
|  |  | | |  |  |  |  |  |  |  |  |
| **Engagement** | The public | | | interacted | The public interacted |  | The public interacted |  | No public |  |  |
|  | significantly | | | with the | with the |  | significantly with the |  | interactions were | |  |
|  | material/student. | | | | material/student. |  | material/student a bit. |  | apparent. Students | |  |
|  | Students excelled in | | | | Students were good at |  | Students answered |  | didn’t answer any | |  |
|  | answering questions if | | | | answering questions if |  | questions if they were |  | questions or it | |  |
|  | they were posed. | | | | they were posed. |  | posed, but answers |  | wasn’t clear if there | |  |
|  | There was a clear plan to | | | | There was a plan to |  | lacked clarity. It wasn’t |  | was a clear plan to | |  |
|  | make the project | | | | make the project |  | clear if there was a |  | make the project | |  |
|  | engaging. | | |  | engaging. |  | clear plan to make the |  | engaging. |  |  |
|  |  |  |  |  |  |  | project engaging. |  |  |  |  |
|  |  | | | |  |  |  |  |  |  |  |
| **Language** | No jargon was used. | | | | Occasional jargon |  | Frequent jargon, not |  | A general | audience |  |
|  |  |  |  |  | and/or explained |  | explained |  | would be | unable to |  |
|  |  |  |  |  | jargon |  |  |  | understand. | |  |
|  |  | | |  |  |  |  |  |  | |  |
| **Execution** | Outreach was | | |  | Outreach was well |  | Average execution. |  | Execution flawed. | |  |
|  | excellently executed. | | | | executed. Outreach |  | Outreach goals mostly |  | Outreach goals either | |  |
|  | Outreach goals were | | | | goals were articulated |  | articulated and |  | not articulated or not | |  |
|  | clearly articulated and | | | | and mostly met. |  | somewhat met. The |  | met. The importance | |  |
|  | met. Target audience | | | | Target audience was |  | importance statement |  | statement was not | |  |
|  | was successfully | | | | mostly reached. There |  | was ok, but not |  | understandable or was | |  |
|  | reached. There was a | | | | was a clear statement |  | impactful. |  | not made. |  |  |
|  | compelling statement | | | | about why the topic |  |  |  |  |  |  |
|  | about why the topic was | | | | was important. |  |  |  |  |  |  |
|  | important. | | |  |  |  |  |  |  |  |  |
| **Overall** | Any supporting | | | | Any supporting |  | Any supporting |  | Any supporting | |  |
| **aesthetics** | materials, images, art, | | | | materials, images, art, |  | materials, images, art, |  | materials, images, art, | |  |
|  | tone, etc. successfully | | | | tone, etc. mostly |  | tone, etc. sometimes |  | tone, etc. often did not | |  |
|  | enhanced the outreach. | | | | enhanced the |  | enhanced the outreach. |  | enhance the outreach. | |  |
|  | Credit was given for | | | | outreach. Credit was |  | Credit was given for |  | Credit not given for | |  |
|  | anything used that | | | | given for anything |  | most things used that |  | things used that | |  |
|  | wasn’t the student’s. | | | | used that wasn’t the |  | weren’t the student’s. |  | weren’t the student’s. | |  |
|  | Correct grammar/ | | | | student’s. Correct |  | Correct grammar/ |  | Major grammatical/ | |  |
|  | spelling was used | | | | grammar/spelling was |  | spelling was sometimes |  | spelling errors were | |  |
|  | throughout. Length | | | | used mostly |  | used. Length was either |  | made. Length was | |  |
|  | kept people interested | | | | throughout. Length |  | too long or too short. |  | either too long or too | |  |
|  | and engaged. | | |  | mostly kept people |  |  |  | short. |  |  |
|  |  |  |  |  | interested and |  |  |  |  |  |  |
|  |  |  |  |  | engaged. |  |  |  |  |  |  |
|  |  |  |  |  |  |  |  |  |  |  |  |
