# Supplementary material for: Establishing a baseline of science communication skills in an undergraduate environmental science course
Source: Int J STEM Educ. 2021 Jul 23;8(1):47. doi: 10.1186/s40594-021-00304-0 (PMC8299166; doi:10.1186/s40594-021-00304-0)
Supplement: Supplementary file 3 — Additional file S3: Codebook [file 40594_2021_304_MOESM3_ESM.docx]

# Instructions for all coding:

Elements must be explicitly stated. Do not assume, read between the lines, or overreach what is written.

# What elements do students address in their projects (presence)?

Projects (i.e., plans and products) were analysed for the presence of each of the 13 elements of the framework. For example, a plan that included descriptions of the audience, communication objectives (purpose), and scientific content received 1s for those elements to identify the presence of those elements. But the plan received 0s for the remaining elements because it lacked information on collecting prior knowledge, identifying how language would be used, identifying what stylistic components would be used, how the presentation would appeal to the senses, how the audience would engage with the science, how the student would facilitate a dialogue with the audience, how the product would be delivered (mode), and where it would be delivered (platform). Below are further descriptions of what constitutes presence for each element.

| **Elements of SciComm** | **Descriptions of Element Presence:** Codes are separated into two cells by their specificity on plans and products. Codes that are applicable to both plans and products will be described in one cell. | |
| --- | --- | --- |
|  | Codes specific to **Plans** | Codes specific to **Products** |
| **Audience** | Present if students described who they planned to communicate with. | Present if students shared their product with an audience. |
|  | Emergent thematic analysis of student plans and products generated three themes for how students addressed the element of audience: the age of the intended audience, interest, and specificity of the described audience. Plans and products were coded not applicable (NA) if the audience characteristic was unknown because it was not identified, unseen, or unable to be determined (e.g., cannot determine age based on a social media comment). Age The age of the audience was coded as child, young adult, or adult.   - A plan or product was coded as **child** if the audience were in elementary school (5^th^ grade or younger). - A plan or product was coded as **young adult** if the audience was in middle or high school (6^th^-12^th^ grade). (The words “young adult” could be used in a descriptor, but not be coded as such, if the intended audience is another age, such as in college.) - A plan or product was coded as **adult** if the audience was in college or out of school. - Each plan could receive multiple codes for audience age, if appropriate. For example, some students described multiple, different audiences. - Plans could receive no codes for audience age if they did not explicitly describe their audience. For example, “general public” or posting to “social media” were too non-specific to code for audience age.  Interest The interest of the audience was coded as interested or uninterested.   - A plan was coded as **interested** if the audience had explicit or implicit interest in the subject matter. For example, “seasoned naturalists” implicitly are interested in top predators and trophic levels. An audience could be coded as interested even if they “do not believe” the science such as anti-vaccinators. - A plan was coded as **uninterested** if the audience had no interest in the subject. Further, an audience was coded as uninterested if they discussed things like “did not notice” or “doesn’t make a difference” because although they had some knowledge of the topic, they were not interested in learning about it or applying it. - A lack of knowledge also did NOT assume a lack of interest, if this was unclear, the plans received no code for interest. - Each plan could receive multiple codes for audience interest, if appropriate. - Plans could receive no codes for audience interest if they did not explicitly describe the presence of interest in their audience.  Specificity The specificity of the audience was coded as general or specific.   - A plan was coded **general** when it included a description of a more general audience (e.g., “public,” “people,” “wide audience”). A product was coded general if the product was shared publicly for example, on a public social media site. - A plan was coded **specific** when it included a description of a more specific audience commonly in relation to age, interest, awareness, or knowledge (or lack of knowledge). A product was coded specific when a narrowed audience was observable such as a classroom or private social media post. - Plans could be coded as **both** general and specific if there were explicit audience descriptions and very vague, general statements about a wider audience. Products could be coded for both if it were presented to a private audience and displayed publicly. - Plans could receive no codes for audience specificity if they did not explicitly describe audience specificity. | |
| **Prior Knowledge** | Present if students described instances of researching background information on their audience or asking questions prior to presenting information. | The element of prior knowledge was not observable in products. |
|  | Emergent thematic analysis of student plans and products generated two themes for how students addressed the element of prior knowledge: the collection of prior knowledge and the use of prior knowledge.   - Student plans were coded for **collection** of prior knowledge if students planned to consider what information their audience knew before presenting their information. This could be as research done before the doing the product or questions asked at the beginning (but not the end) of a presentation. Prior knowledge could be collected on the exact population that will serve as the audience or a representative sample or similar population as a future audience. Collecting prior knowledge could include students performing surveys themselves or using results from reliable sources. The results of prior knowledge may or may not (although should) impact planned execution of the project. - Student plans were coded for **use** of prior knowledge when students both collected prior knowledge about their audience *and* used or planned to use the information gathered to influence their project. For example, asking an audience for what they know about a topic before a presentation *and* tailoring the presentation to misconceptions revealed by the question. A 0 (no) for prior knowledge use indicates that the presentation would have occurred as planned with or without collecting the prior knowledge. | |
| **Purpose: Objectives** | Present if students described their goals for the product, or what the student hoped to accomplish with the product. | The element of purpose was not observable in products. |
|  | In the assignment instructions, students were prompted to create a list of goals for their SciComm product and what they hoped to accomplish. We analyzed the science communication objectives that students described in their plans using *a priori* thematic analysis. Specifically, plans were coded for the following science communication objectives: increase knowledge and awareness, boost interest and excitement, listen and demonstrate openness, prove competence, reframe issues, impart shared values, and convey warmth and respect. In addition to these objectives, we identified other common objectives, such as explicitly aiming to have the audience take action based on the product.   - **Increase awareness and knowledge** – educate the audience on specific issues, topics, or scientific processes to provide a better understanding, correct misconceptions, change thinking, fill a gap in knowledge, or simply share what the student knows or cares about (but the student must be objective with the audience). - **Convey competence** – establish why the communicator is qualified to speak about the topic with authority (also useful to gain the trust of the audience). - **Boost interest and excitement** – being interesting and entertaining for the purpose of getting the audience excited about the science. - **Listen and demonstrate openness** – show the audience that the student is willing to listen and may incorporate audience input. - **Reframe issues** – provide the context of the scientific information in a way that resonates with the audiences' views. Relevance between audience and science (not necessarily the student). - **Convey shared values** – respect the values and identity of the audience, and students must be open about their own values and identity. Maybe try to find common values or identity between students and audience (not necessarily commonalities with the science). - **Convey warmth and respect** – be genuine, friendly, helpful, sincere, caring, compassionate, honest, and moral. - **Action** – aim to have the audience act because of the SciComm (e.g., recycle better). - **Absent** - No communication objectives are defined explicitly. Even if students mention their "goal" it commonly just describes what they will do (e.g., "show the audience this and that") rather than what they are hoping to accomplish. - Each plan could receive multiple objective codes, if appropriate. | |
| **Theory** | Present if students described a theoretical reason or rationale for planning the project a certain way (or specific element in the project such as the content or picking the audience). May or may not be accurate or evidence-based theoretical reasoning or rationale. | The element of theory was not observable in products. |
| **Content** | Present if students described what scientific content they intended to communicate with their audience. | Present if students submitted a product that included scientific information. |
|  | Emergent thematic analysis of student plans and products generated two themes for how students addressed the element of scientific content: the components and the topics of the content.  *Components*  Emergent thematic analysis of student plans and products generated two themes for how students addressed the element of scientific content: human components and biological (non-human) components.   - The content was coded as **human** if it included explicit human interactions with the environment such as choices that directly affect the environment (e.g., decisions that ultimately affect other species); it included implicit ideas of how humans can “help” or “get involved” with an issue. - The content was coded as **biological** if it included information about non-human species, their interactions, and their importance (e.g., predators, agriculture, food webs). A plan included only vague terms like “environment”; it would not be coded as biological. - Multiple codes were assigned for components, if applicable.  Topics Initially, the instructor directed the students to do their projects on the other project topics in the course (sustainability, ecology, climate change) but later encouraged them to pick anything they were excited about. We used the identified topics for *a priori* thematic analysis and included another code for topics that did not fit these.   - The content was coded as **sustainability** if it included information about any topic that related to helping the environment to become more sustainable (e.g., recycling, alternative agriculture, alternative energy sources, etc.). If it was only about saving/protecting species, then it was not code as sustainability. - The content was coded as **ecology** if it included information about ecosystems, food webs, top predators, conservation of species, etc. If a plan only included information about a species and its uses for human consumption (e.g., the plants used in alternative agriculture) it was not coded as ecology. - The content was coded as **climate change** if it included information about climate change in general, weather issues related to climate change, or human health issues associated with climate change. - The content was coded as **other** if it did not fit any other aforementioned topics codes. These included the topics of oil spills, weather—not explicitly climate change-related— plastics and included information about effects of pollution on humans and other organisms without specifying the effects on the food web. - Multiple codes were assigned for the topic, if applicable. | |
| **Context** | Present if students described that the environmental issue (content) is situated within social, cultural, and or political contexts. For example, a student describes how climate change is harmful to human health, yet it’s culturally acceptable to drive to a store a few blocks away; or that overfishing negatively affects marine populations, yet it’s been some people's livelihoods (social) for generations (cultural). Could be thought of as providing the “flip side” or why humans are “causing” negative environmental issues. Likewise, students could also present information on how the environmental issues affect the social, cultural, or political factors. The context must be explicit and not assumed; if the student just mentions a component that relates to context (e.g., climate change, overfishing) but doesn’t explain its relationships to social, cultural, or political factors, then it’s coded as a 0. Also, the presence of context is not just the presence of a human component to the scientific issue (that is covered in content components: human and does not describe the possible reasons).  We used *a prior* analysis to identify how students addressed the element of context that included the social, cultural, and or political contexts. However, too few plans and products included context to delineate further. Additionally, no other prevalent themes emerged organically. | |
| **Dialogue** | Present if students described having any exchange to, from, or with their audience. The conversation could be on any topic and not just about the scientific content. | Present if students shared something with an audience. |
|  | Student plans and products were analysed for the element of dialogue in two ways: the direction of the communication and level of dialogue. Direction The direction of dialogue was could be categorized as   1. from student to audience only, 2. from audience to student only, or 3. both to and from the audience and student.  Level The level of dialogue was categorized as low, medium, and high. One category was assigned for each plan and product.   - **Low** dialogue was when only one direction of communication was planned or occurred (e.g., student to audience only). - **Medium** dialogue was when both directions of dialogue were planned or occurred, but one direction occurred much more frequently than the other (e.g., presentation with a brief Q&A session). - **High** dialogue was when both directions of dialogue were planned or occurred frequently. This dialogue does not have to occur between student and the audience, the dialogue can be between members of the audience. | |
| **Engagement** | Present if students described if or how the audience would interact with the science. | Present if there was evidence that the students had the audience interact with the science. |
|  | Student projects were analysed for engagement between the audience and the science in two ways: the type of engagement and level of engagement.  *Type*  Emergent thematic analysis of student plans and products generated four themes for how students addressed the element of engagement: passive, questioning, active, and ambiguous.   - A plan or product was coded as **passive** engagement when the student described that engagement between the audience and science will be passive and not require or show evidence that the audience is doing or thinking about the science, such as listening, viewing what the student is presenting, lecturing, showing, explaining, or demonstrating. - A plan or product was coded as **active** engagement when the student described how the product will show evidence of the audience interacting with the science such as the audience modelling a scientific phenomenon and taking part in scientific inquiry (not including observing a lecture or demonstration). - A plan or product would not be coded as active or passive if the audience is simply taking part in a survey unless there was explicit discussion about the scientific content. - A plan or product was coded for a question if there was an exchange of **questions** about the scientific content with the audience.   - A plan or product was coded as **questions from student** engagement when the student described that engagement between the audience and science will be through asking **specific** questions about the science to the audience. (For example, it could include having the audience answer trivia or survey questions.   - A plan or product was coded as **questions/comments from audience** engagement when the student described that engagement between the audience and science will include getting questions or comments from the audience. This includes questions in a live questioning as well as questions through a social media forum. A product was not coded as questions/comments from the audience when the audience was simply answering a question in a survey or trivia format. - A plan was coded as **ambiguous** engagement when the student described that engagement between the audience and science will occur but does not describe how. Products could not be coded as ambiguous. - Each plan or product could be coded as having multiple types of engagement.   *Level*  The level of engagement was categorized as low, medium, and high. One category was assigned for each plan and product, even if there was an absence of engagement.   - A plan or product was categorized as **low** engagement when the student was presenting, and the audience was listening or viewing for the entire time or if there was no engagement (absence). - A plan or product was categorized as **medium** engagement when the student was presenting and the audience listening or viewing *most of the time*, but the communication also included some interaction between the audience and the science, commonly as questions or comments from the audience. - A plan or product was categorized as **high** engagement when the student facilitated active and frequent interaction between the audience and the science, such as the audience answering specific questions and modelling a scientific phenomenon or taking part in scientific inquiry. | |
| **Language** | Present if students described what specific language they would or would not include such as jargon, technical works, discipline-specific words, colloquial phrasing, formal wording, etc. | Present if students used specific language such as jargon, technical works, discipline-specific words, colloquial phrasing, formal wording, etc. |
|  | Emergent thematic analysis of student plans and products generated two themes for how students addressed the element of language: the use of jargon and formality. Plans and products could receive more than one code. For example, a product could be coded as both formal (F) and using jargon (J). Plans were only coded or categorized for language if a student described the planned, intentional use of language in the product; therefore, many plans did not receive a code for language. Jargon Categories  - **Jargon** (J): Student uses technical terms to describe the product, and the audience would have difficulty understanding without knowledge of the topic. - **No Jargon** (NJ): Student does not use technical terms during the whole project. - A plan or product could only be categorized as either jargon or no jargon, not both.  Formality Codes  - **Formal** Language (F): Student uses language and tone that would be used in a professional setting. For example, a student “presenting” to a class would be coded as formal. - **Colloquial** Language (C): Student uses informal, everyday language and tone that would be used in a casual conversation. - A product could be coded for both formal and colloquial language if the presentation had various components at various times and would be categorized as **mixed** formality. | |
| **Mode** | Present if students described what they would use to share their scientific information (commonly as media). | Present if students used some form of media to share their scientific information. |
|  | Emergent thematic analysis of student plans and products generated three themes for how students addressed the element of mode: the **location** of the product (either in-person or remote) and the **type of media** used (audio, print, or video).  *Location*  The location was coded as in-person or remote.   - A plan or product was coded as **in-person** when the person was present to interact with the audience (regardless of the type of media used). - A plan or product was coded as **remote** when the project was posted digitally (various online websites) or the material was posted, and the communicator left the location (no in-person interaction). - Each plan could receive multiple codes for mode location, if appropriate.  Type The type of media was coded as audio, print, or video.   - A plan or product was coded as **audio** when the project included an audio recording. Verbal discussion is not included here without the use of media. Products were not coded for audio if a student was presenting and simply talking to the audience. - A plan or product was coded as **print** when the project included some form of static visual. Static visuals included in a video product would not receive a “print” code, only a video code. Simply posting questions/comments on social media is not a form of print media. - A plan or product was coded as **video** when the project included some form of video presentation or recording (regardless of if it was used in-person or posted digitally). Video media usually includes an audio component, but this is encompassed in the video code and would not require separate coding in audio media. - Each plan and product could receive multiple codes for mode type, if appropriate, or no codes if they were not using media. | |
| **Platform** | Present if students described where they planned to share or host their product (the setting they planned to use to share or host their product). | Present if students shared or hosted their product beyond just submitting the assignment to the instructor. |
|  | Emergent thematic analysis of student plans and products generated a single theme for how students addressed the element of platform: the use of social media or not.   - If the student stated in their plan that they were using social media (1), the platform of social media they planned to use was recorded (e.g., Twitter, Facebook, Instagram). - If the student was not using social media (0), any other platform details were recorded (e.g., classroom, gym). - Products were coded for if they were posted on social media as a part of the project, not necessarily if only posted privately to submit the project (e.g., YouTube to share large video files). | |
| **Style** | In plans and products, the project was coded for style if the student described or used a stylistic element such as humour, anecdotes, analogies, metaphors, rhetoric, storytelling, and narratives. This does not include sharing information (e.g., data) if they do not use a stylistic element to do so. We used the aforementioned types of style for *a prior* analysis, but no type of style was present in enough frequency to delineate further. Additionally, no other themes appeared organically. | |
| **Appeal** | Present if students described the use of appeal of the audience’s senses. Usually related to the five traditional senses in humans: sight, hearing, taste, smell, and touch. Includes use of visuals aids, eye contact, body language, use of music, and tone of voice. In the product, if the student made a clear effort to make the project visually or auditory appealing, then the project was coded for appeal.  We used priori thematic analysis to identify which senses students planned to appeal to and appealed to during presentations using the five traditional senses in humans. Common examples of appealing to each sense are also listed below.   - **Sight** – Visuals aids, eye contact, body language. Does not include text such as comments that go along with social media postings or survey questions, unless the student intentionally uses clear or interesting font styles or sizes. - **Hearing** – Use of music and tone of voice. Does not include the student just describing talking to the audience, whether in person or recorded, unless specific elements of the oration are used, such as intentional tone of voice. - **Taste** – No examples of student work. - **Smell** – No examples of student work. - **Touch** – Manipulate rocks. | |

# What elements do the assignment instructions and rubric request students to include?

The assignment instructions and rubric were analysed for which elements of the framework were explicitly requested. The assignment instructions and rubric were analysed for if they requested each element of the framework using the same general codebook as described above for presence of elements in student work.
